# Crystal structure of dimanganese(II) zinc bis­[ortho­phosphate(V)] monohydrate

**DOI:** 10.1107/S2056989015000341

**Published:** 2015-01-14

**Authors:** Ghaleb Alhakmi, Abderrazzak Assani, Mohamed Saadi, Lahcen El Ammari

**Affiliations:** a Laboratoire de Chimie du Solide Appliquée, Faculté des Sciences, Université Mohammed V, Avenue Ibn Battouta, BP 1014, Rabat, Morocco

**Keywords:** crystal structure, transition metal phosphates, hydro­thermal synthesis, Fe_3_(PO_4_)_2_·H_2_O structure type

## Abstract

The title compound, Mn_2_Zn(PO_4_)_2_·H_2_O, was obtained under hydro­thermal conditions. The structure is isotypic with other transition metal phosphates of the type *M*_3−_ *_x_M*′_*x*_(PO_4_)_2_·H_2_O, but shows no statistical disorder of the three metallic sites. The principal building units are distorted [MnO_6_] and [MnO_5_(H_2_O)] octa­hedra, a distorted [ZnO_5_] square pyramid and two regular PO_4_ tetra­hedra. The connection of the polyhedra leads to a framework structure. Two types of layers parallel to (-101) can be distinguished in this framework. One layer contains [Zn_2_O_8_] dimers linked to PO_4_ tetra­hedra *via* common edges. The other layer is more corrugated and contains [Mn_2_O_8_(H_2_O)_2_] dimers and [MnO_6_] octa­hedra linked together by common edges. The PO_4_ tetra­hedra link the two types of layers into a framework structure with channels parallel to [101]. The H atoms of the water mol­ecules point into the channels and form O—H⋯O hydrogen bonds (one of which is bifurcated) with framework O atoms across the channels.

## Abstract 2

In contrast to most other structures of transition metal orthophosphates with composition *M*_3-_ *_x_M*’_*x*_(PO_4_)_2_·H_2_O, the three metallic sites in the structure of Mn_2_Zn(PO_4_)_2_·H_2_O show no statistical disorder.

## Chemical context   

The great structural diversity of metal-based phosphates, associated with their physical properties makes this family of compounds inter­esting as potential functional materials, *e.g.* as catalysts (Viter & Nagornyi, 2009 ▸; Weng *et al.*, 2009 ▸) or ion-exchangers (Jignasa *et al.*, 2006 ▸). Among the wide variety of metal phosphates, one of our inter­ests is focused on mixed metallic orthophosphates of general formula *M*_3−_ *_x_M′_x_*(PO_4_)_2_·H_2_O. The present communication reports the hydro­thermal synthesis and structural characterization of a new member of this family, Mn_2_Zn(PO_4_)_2_·H_2_O.

## Structural commentary   

**Figure 1:**
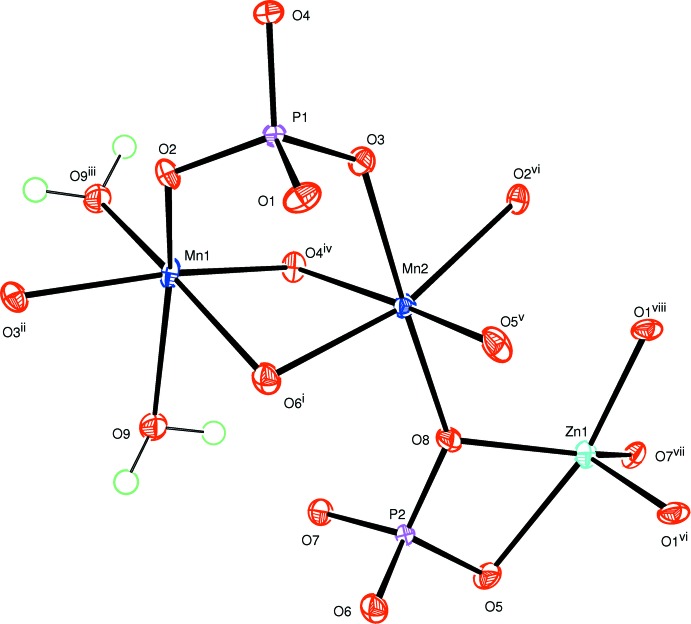
The principal building units in the structure of Mn_2_Zn(PO_4_)_2_·H_2_O. Displacement ellipsoids are drawn at the 50% probability level. Hydrogen bonds are indicated by dashed lines. [Symmetry codes: (i) −*x* + 1, −*y* + 1, −*z* + 1; (ii) *x* + 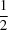, −*y* + 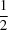, *z* + 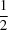; (iii) −*x* + 2, −*y* + 1, −*z* + 1; (iv) −*x* + 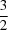, *y* + 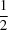, −*z* + 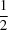; (v) −*x* + 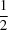, *y* − 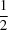, −*z* + 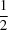; (vi) *x* − 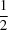, −*y* + 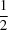, *z* − 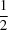; (vii) *x* − 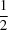, −*y* + 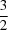, *z* − 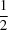; (viii) −*x* + 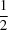, *y* + 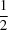, −*z* + 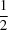.]

**Figure 2:**
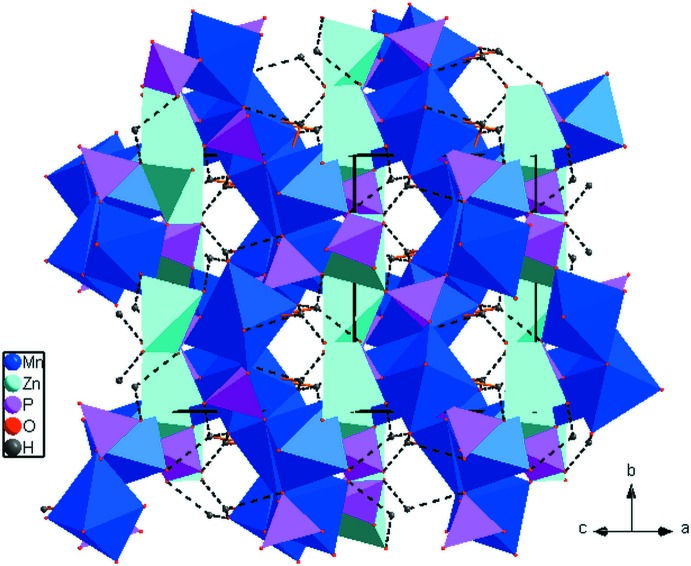
Polyhedral representation of Mn_2_Zn(PO_4_)_2_·H_2_O showing channels extending parallel to [101]. Hydrogen bonds are shown as dashed lines.

The structure of the title compound is built up from four different types of building units: [MnO_6_] and [MnO_5_(H_2_O)] octa­hedra, [ZnO_5_] square pyramids and PO_4_ tetra­hedra, as shown in Fig. 1 ▸. Whereas the [MnO_6_] octa­hedron is more or less regular with Mn—O distances in the range 2.1254 (13) to 2.2590 (13) Å, the [MnO_5_(H_2_O)] octa­hedron is significantly distorted with five equal Mn—O distances in the range 2.1191 (13) to 2.1556 (16) and one considerably longer Mn—O distance to the water ligand of 2.5163 (15) Å; the ZnO_5_ square pyramid is also distorted with four shorter Zn—O distances between 1.9546 (13) and 2.0347 (12) Å and one longer Zn—O distance, likewise to the water O atom [2.3093 (14) Å]; the two PO_4_ tetra­hedra are rather regular [P—O distances between 1.5322 (13) and 1.5570 (13) Å; O—P—O angles between 102.92 (7) and 111.62 (8)°]. These polyhedra are arranged in such a way as to build up two types of layers parallel to (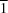
01). One layer contains two [ZnO_5_] polyhedra linked together by edge-sharing into a [Zn_2_O_8_] dimer that in turn is linked to PO_4_ tetra­hedra. The other layer contains dimers of the type [Mn_2_O_8_(H_2_O)_2_] (also formed by edge-sharing of two [MnO_5_(H_2_O)] octa­hedra), connecting [MnO_6_] octa­hedra and PO_4_ tetra­hedra through common vertices. The two types of layers are linked by common edges and vertices into a framework structure with channels parallel to [101]. The water mol­ecules of the [MnO_5_(H_2_O)] octa­hedra protrude into these channels and develop hydrogen bonds (one bifurcated) of medium-to-weak strength to framework O atoms across the channels (Fig. 2 ▸; Table 1 ▸).

The title compound adopts the Fe_3_(PO_4_)_2_·H_2_O structure type (Moore & Araki, 1975 ▸) and is isotypic with various structures of general formula *M*_3−_ *_x_M′_x_*(PO_4_)_2_·H_2_O: CuMn_2_(PO_4_)_2_·H_2_O (Liao *et al.*, 1995 ▸); Co_2.59_Zn_0.41_(PO_4_)_2_·H_2_O (Sørensen *et al.*, 2005 ▸); Co_2.39_Cu_0.61_(PO_4_)_2_·H_2_O (Assani *et al.*, 2010 ▸); Mg_1.65_Cu_1.35_(PO_4_)_2_·H_2_O (Khmiyas *et al.* 2015 ▸).

## Synthesis and crystallization   

Crystals of Mn_2_Zn(PO_4_)_2_·H_2_O were obtained by hydro­thermal treatment of zinc oxide (0.0406 g), metallic manganese (0.0824 g), phospho­ric acid (0.1 ml) and 12.5 ml of distilled water, in a proportion corresponding to the molar ratio Zn: Mn: P = 1: 3: 3. The hydro­thermal reaction was conducted in a 23 ml Teflon-lined autoclave under autogenous pressure at 493 K for five days. After being filtered, washed with deionized water and dried in air, the reaction product consisted of two types of crystals, the first as off-white parallelepipeds corresponding to Mn_7_(PO_4_)_2_(HPO_4_)_4_ (Riou *et al.*, 1987 ▸) and the second as colourless parallelepipeds corres­ponding to the title compound.

## Refinement   

Crystal data, data collection and structure refinement details are summarized in Table 2 ▸. The O-bound H atoms were initially located in a difference map. In the last refinement cycle the distances were fixed at 0.89 and 0.91 Å, respectively, and the H atoms refined in the riding-model approximation with *U*_iso_(H) set to 1.5*U*_eq_(O). The highest peak and the deepest hole in the final Fourier map are at 0.32 Å and 0.30 Å, respectively, from Mn1 and Zn1.

## Supplementary Material

Crystal structure: contains datablock(s) I. DOI: 10.1107/S2056989015000341/wm5102sup1.cif

Structure factors: contains datablock(s) I. DOI: 10.1107/S2056989015000341/wm5102Isup2.hkl

CCDC reference: 1042563

Additional supporting information:  crystallographic information; 3D view; checkCIF report

## Figures and Tables

**Table 1:** Hydrogen-bond geometry (, )

*D*H*A*	*D*H	H*A*	*D* *A*	*D*H*A*
O9H1O7	0.89	1.97	2.7866(19)	151
O9H2O5^i^	0.91	2.16	2.8687(19)	134
O9H2O1^ii^	0.91	2.48	3.0494(19)	120

Symmetry codes: (i) 
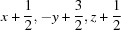
; (ii) 
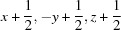
.

**Table 2:** Experimental details

Crystal data	
Chemical formula	Mn_2_Zn(PO_4_)_2_H_2_O
*M*_r_	383.21
Crystal system, space group	Monoclinic, *P*2_1_/*n*
Temperature (K)	296
*a*, *b*, *c* ()	8.1784(2), 10.1741(2), 9.0896(2)
()	114.142(1)
*V* (^3^)	690.17(3)
*Z*	4
Radiation type	Mo *K*
(mm^1^)	7.54
Crystal size (mm)	0.32 0.27 0.19

Data collection	
Diffractometer	Bruker X8 *APEX*
Absorption correction	Multi-scan (*SADABS*; Bruker, 2009 ▸)
*T*_min_, *T*_max_	0.574, 0.748
No. of measured, independent and observed [*I* > 2(*I*)] reflections	11327, 2407, 2305
*R*_int_	0.023
(sin /)_max_ (^1^)	0.746

Refinement	
*R*[*F* ^2^ > 2(*F* ^2^)], *wR*(*F* ^2^), *S*	0.019, 0.052, 1.11
No. of reflections	2407
No. of parameters	127
H-atom treatment	H-atom parameters constrained
_max_, _min_ (e ^3^)	0.94, 0.84

Computer programs: *APEX2* and *SAINT* (Bruker, 2009 ▸), *SHELXS97* and *SHELXL97* (Sheldrick, 2008 ▸), *ORTEP-3 for Windows* (Farrugia, 2012 ▸), *DIAMOND* (Brandenburg, 2006 ▸) and *publCIF* (Westrip, 2010 ▸).

## supplementary crystallographic information

### Crystal data

**Table:**

Mn_2_Zn(PO_4_)_2_·H_2_O	*F*(000) = 736
*M**_r_* = 383.21	*D*_x_ = 3.688 Mg m^−^^3^
Monoclinic, *P*2_1_/*n*	Mo *K*α radiation, λ = 0.71073 Å
Hall symbol: -P 2yn	Cell parameters from 2407 reflections
*a* = 8.1784 (2) Å	θ = 2.8–32.0°
*b* = 10.1741 (2) Å	µ = 7.54 mm^−^^1^
*c* = 9.0896 (2) Å	*T* = 296 K
β = 114.142 (1)°	Parallelepiped, off-white
*V* = 690.17 (3) Å^3^	0.32 × 0.27 × 0.19 mm
*Z* = 4	

### Data collection

**Table:**

Bruker X8 APEX diffractometer	2407 independent reflections
Radiation source: fine-focus sealed tube	2305 reflections with *I* > 2σ(*I*)
Graphite monochromator	*R*_int_ = 0.023
φ and ω scans	θ_max_ = 32.0°, θ_min_ = 2.8°
Absorption correction: multi-scan (*SADABS*; Bruker, 2009)	*h* = −12→12
*T*_min_ = 0.574, *T*_max_ = 0.748	*k* = −14→15
11327 measured reflections	*l* = −13→13

### Refinement

**Table:**

Refinement on *F*^2^	Primary atom site location: structure-invariant direct methods
Least-squares matrix: full	Secondary atom site location: difference Fourier map
*R*[*F*^2^ > 2σ(*F*^2^)] = 0.019	Hydrogen site location: inferred from neighbouring sites
*wR*(*F*^2^) = 0.052	H-atom parameters constrained
*S* = 1.11	*w* = 1/[σ^2^(*F*_o_^2^) + (0.0248*P*)^2^ + 1.0141*P*] where *P* = (*F*_o_^2^ + 2*F*_c_^2^)/3
2407 reflections	(Δ/σ)_max_ = 0.001
127 parameters	Δρ_max_ = 0.94 e Å^−^^3^
0 restraints	Δρ_min_ = −0.84 e Å^−^^3^

### Special details

**Table:**

Geometry. All e.s.d.'s (except the e.s.d. in the dihedral angle between two l.s. planes) are estimated using the full covariance matrix. The cell e.s.d.'s are taken into account individually in the estimation of e.s.d.'s in distances, angles and torsion angles; correlations between e.s.d.'s in cell parameters are only used when they are defined by crystal symmetry. An approximate (isotropic) treatment of cell e.s.d.'s is used for estimating e.s.d.'s involving l.s. planes.
Refinement. Refinement of *F*^2^ against all reflections. The weighted *R*-factor *wR* and goodness of fit *S* are based on *F*^2^, conventional *R*-factors *R* are based on *F*, with *F* set to zero for negative *F*^2^. The threshold expression of *F*^2^ > 2σ(*F*^2^) is used only for calculating *R*-factors(gt) *etc*. and is not relevant to the choice of reflections for refinement. *R*-factors based on *F*^2^ are statistically about twice as large as those based on *F*, and *R*- factors based on all data will be even larger.

### Fractional atomic coordinates and isotropic or equivalent isotropic displacement parameters (Å^2^)

**Table:**

	*x*	*y*	*z*	*U*_iso_*/*U*_eq_	
Mn1	0.88638 (3)	0.35884 (3)	0.46580 (3)	0.00768 (6)	
Mn2	0.48057 (3)	0.38305 (3)	0.21880 (3)	0.00726 (6)	
Zn1	0.12609 (3)	0.62028 (2)	0.06179 (2)	0.00934 (6)	
P1	0.70438 (5)	0.08456 (4)	0.32706 (5)	0.00553 (8)	
P2	0.38560 (5)	0.67442 (4)	0.36388 (5)	0.00613 (8)	
O1	0.58212 (17)	0.03301 (13)	0.40831 (15)	0.0102 (2)	
O2	0.87050 (16)	0.15076 (13)	0.45546 (15)	0.0094 (2)	
O3	0.59293 (17)	0.18429 (12)	0.19887 (15)	0.0092 (2)	
O4	0.76145 (17)	−0.03217 (12)	0.25178 (15)	0.0092 (2)	
O5	0.23736 (18)	0.77279 (13)	0.26723 (16)	0.0123 (2)	
O6	0.36400 (17)	0.63194 (13)	0.51688 (15)	0.0104 (2)	
O7	0.57269 (16)	0.73311 (13)	0.41028 (15)	0.0110 (2)	
O8	0.35411 (17)	0.55914 (13)	0.24330 (15)	0.0107 (2)	
O9	0.88135 (18)	0.58568 (14)	0.57419 (16)	0.0130 (2)	
H1	0.7876	0.6203	0.4923	0.019*	
H2	0.8793	0.5969	0.6731	0.019*	

### Atomic displacement parameters (Å^2^)

**Table:**

	*U*^11^	*U*^22^	*U*^33^	*U*^12^	*U*^13^	*U*^23^
Mn1	0.00519 (11)	0.00776 (11)	0.00860 (11)	−0.00070 (8)	0.00129 (8)	0.00274 (8)
Mn2	0.00657 (11)	0.00704 (11)	0.00777 (11)	−0.00016 (8)	0.00254 (9)	−0.00025 (8)
Zn1	0.00839 (10)	0.00949 (10)	0.00884 (9)	0.00028 (6)	0.00219 (7)	0.00093 (6)
P1	0.00538 (16)	0.00594 (17)	0.00519 (16)	0.00046 (13)	0.00209 (13)	−0.00030 (13)
P2	0.00556 (16)	0.00676 (17)	0.00569 (16)	0.00008 (13)	0.00193 (13)	−0.00044 (13)
O1	0.0107 (5)	0.0111 (5)	0.0126 (5)	0.0015 (4)	0.0087 (5)	0.0025 (4)
O2	0.0079 (5)	0.0105 (5)	0.0073 (5)	−0.0020 (4)	0.0008 (4)	−0.0025 (4)
O3	0.0099 (5)	0.0080 (5)	0.0081 (5)	0.0022 (4)	0.0020 (4)	0.0013 (4)
O4	0.0083 (5)	0.0091 (5)	0.0099 (5)	0.0017 (4)	0.0033 (4)	−0.0027 (4)
O5	0.0121 (5)	0.0135 (6)	0.0108 (5)	0.0070 (5)	0.0041 (4)	0.0035 (4)
O6	0.0106 (5)	0.0137 (6)	0.0071 (5)	−0.0006 (4)	0.0038 (4)	0.0016 (4)
O7	0.0081 (5)	0.0129 (6)	0.0120 (5)	−0.0031 (4)	0.0041 (4)	−0.0030 (4)
O8	0.0107 (5)	0.0093 (5)	0.0099 (5)	0.0011 (4)	0.0021 (4)	−0.0035 (4)
O9	0.0109 (5)	0.0181 (6)	0.0106 (5)	0.0020 (5)	0.0051 (4)	0.0007 (5)

### Geometric parameters (Å, º)

**Table:**

Mn1—O6^i^	2.1191 (13)	Zn1—O1^vi^	2.0242 (13)
Mn1—O2	2.1208 (14)	Zn1—O1^viii^	2.0347 (12)
Mn1—O3^ii^	2.1464 (12)	Zn1—O5	2.3093 (14)
Mn1—O9^iii^	2.1504 (14)	P1—O3	1.5327 (13)
Mn1—O4^iv^	2.1556 (13)	P1—O4	1.5355 (13)
Mn1—O9	2.5163 (15)	P1—O2	1.5377 (13)
Mn2—O8	2.1254 (13)	P1—O1	1.5570 (13)
Mn2—O5^v^	2.1533 (13)	P2—O7	1.5322 (13)
Mn2—O4^iv^	2.1921 (13)	P2—O6	1.5340 (13)
Mn2—O2^vi^	2.2126 (13)	P2—O5	1.5401 (13)
Mn2—O6^i^	2.2166 (13)	P2—O8	1.5532 (13)
Mn2—O3	2.2590 (13)	O9—H1	0.8939
Zn1—O7^vii^	1.9546 (13)	O9—H2	0.9131
Zn1—O8	2.0174 (13)		
			
O6^i^—Mn1—O2	90.23 (5)	O4^iv^—Mn2—O3	87.66 (5)
O6^i^—Mn1—O3^ii^	109.27 (5)	O2^vi^—Mn2—O3	76.87 (5)
O2—Mn1—O3^ii^	81.31 (5)	O6^i^—Mn2—O3	87.34 (5)
O6^i^—Mn1—O9^iii^	161.48 (5)	O7^vii^—Zn1—O8	132.60 (5)
O2—Mn1—O9^iii^	107.27 (5)	O7^vii^—Zn1—O1^vi^	100.19 (6)
O3^ii^—Mn1—O9^iii^	80.06 (5)	O8—Zn1—O1^vi^	99.81 (5)
O6^i^—Mn1—O4^iv^	81.32 (5)	O7^vii^—Zn1—O1^viii^	117.98 (5)
O2—Mn1—O4^iv^	118.14 (5)	O8—Zn1—O1^viii^	107.48 (5)
O3^ii^—Mn1—O4^iv^	158.49 (5)	O1^vi^—Zn1—O1^viii^	80.41 (5)
O9^iii^—Mn1—O4^iv^	84.99 (5)	O7^vii^—Zn1—O5	87.57 (5)
O6^i^—Mn1—O9	76.07 (5)	O8—Zn1—O5	67.61 (5)
O2—Mn1—O9	157.28 (5)	O1^vi^—Zn1—O5	167.20 (5)
O3^ii^—Mn1—O9	86.18 (5)	O1^viii^—Zn1—O5	105.05 (5)
O9^iii^—Mn1—O9	89.00 (5)	O3—P1—O4	111.58 (7)
O4^iv^—Mn1—O9	78.15 (5)	O3—P1—O2	110.68 (7)
O8—Mn2—O5^v^	89.03 (5)	O4—P1—O2	109.99 (7)
O8—Mn2—O4^iv^	98.10 (5)	O3—P1—O1	106.62 (7)
O5^v^—Mn2—O4^iv^	167.53 (5)	O4—P1—O1	108.79 (7)
O8—Mn2—O2^vi^	104.14 (5)	O2—P1—O1	109.08 (7)
O5^v^—Mn2—O2^vi^	90.14 (5)	O7—P2—O6	109.42 (7)
O4^iv^—Mn2—O2^vi^	97.96 (5)	O7—P2—O5	111.62 (8)
O8—Mn2—O6^i^	91.85 (5)	O6—P2—O5	110.15 (7)
O5^v^—Mn2—O6^i^	91.25 (5)	O7—P2—O8	110.32 (7)
O4^iv^—Mn2—O6^i^	78.36 (5)	O6—P2—O8	112.31 (8)
O2^vi^—Mn2—O6^i^	163.97 (5)	O5—P2—O8	102.92 (7)
O8—Mn2—O3	173.90 (5)	H1—O9—H2	114.6
O5^v^—Mn2—O3	84.95 (5)		

Symmetry codes: (i) −*x*+1, −*y*+1, −*z*+1; (ii) *x*+1/2, −*y*+1/2, *z*+1/2; (iii) −*x*+2, −*y*+1, −*z*+1; (iv) −*x*+3/2, *y*+1/2, −*z*+1/2; (v) −*x*+1/2, *y*−1/2, −*z*+1/2; (vi) *x*−1/2, −*y*+1/2, *z*−1/2; (vii) *x*−1/2, −*y*+3/2, *z*−1/2; (viii) −*x*+1/2, *y*+1/2, −*z*+1/2.

### Hydrogen-bond geometry (Å, º)

**Table:**

*D*—H···*A*	*D*—H	H···*A*	*D*···*A*	*D*—H···*A*
O9—H1···O7	0.89	1.97	2.7866 (19)	151
O9—H2···O5^ix^	0.91	2.16	2.8687 (19)	134
O9—H2···O1^ii^	0.91	2.48	3.0494 (19)	120

Symmetry codes: (ii) *x*+1/2, −*y*+1/2, *z*+1/2; (ix) *x*+1/2, −*y*+3/2, *z*+1/2.
